# Comparing Performance and Reliability of Collocated Enhanced Children’s MicroPEM (ECM) on Gravimetric and Nephelometric PM_2.5_ Personal Exposure Samples in Field Measurements in Rural Guatemala

**DOI:** 10.1155/ina/8812602

**Published:** 2025-01-02

**Authors:** Erick Mollinedo, John P. McCracken, Michael Johnson, Ricardo Piedrahita, Ajay Pillarisetti, Lance A. Waller, Jiantong Wang, Lisa M. Thompson, Anaite Diaz-Artiga, Oscar de Leon, Alexander Ramirez, Alejandro Polanco, Devan Campbell, Katherine A. Kearns, Jacob Kremer, Laura Nicolaou, Maggie L. Clark, Kalpana Balakrishnan, Ghislaine Rosa, Jennifer L. Peel, William Checkley, Thomas F. Clasen, Luke P. Naeher

**Affiliations:** 1 Department of Environmental Health Science, College of Public Health, University of Georgia, Athens, Georgia, USA; 2 Global Health Institute, College of Public Health, University of Georgia, Athens, Georgia, USA; 3 Center for Health Studies, Universidad del Valle de Guatemala, Guatemala City, Guatemala; 4 Berkeley Air Monitoring Group, Berkeley, California, USA; 5 Division of Environmental Health Sciences, School of Public Health, University of California, Berkeley, California, USA; 6 Department of Biostatistics and Bioinformatics, Rollins School of Public Health, Emory University, Atlanta, Georgia, USA; 7 Nell Hodgson Woodruff School of Nursing, Emory University, Atlanta, Georgia, USA; 8 Gangarosa Department of Environmental Health, Rollins School of Public Health, Emory University, Atlanta, Georgia, USA; 9 Benchmark Risk Group, Chicago, Illinois, USA; 10 Exponent, Oakland, California, USA; 11 Division of Pulmonary and Critical Care, School of Medicine, Johns Hopkins University, Baltimore, Maryland, USA; 12 Department of Environmental and Radiological Health Sciences, Colorado State University, Fort Collins, Colorado, USA; 13 Department of Environmental Engineering, Sri Ramachandra Institute of Higher Education and Research, Chennai, Tamil Nadu, India; 14 Department of Infectious and Tropical Diseases, London School of Hygiene & Tropical Medicine, London, UK

## Abstract

Traditional gravimetric instruments used for personal air pollution exposure measurements are often cumbersome and noisy and do not offer real-time assessment capability. The Enhanced Children’s MicroPEM (ECM) is a comparatively lightweight and quiet instrument designed to capture both integrated filter-based gravimetric samples and real-time continuous (nephelometric) particulate matter with an aerodynamic diameter of 2.5 *μ*m or less (PM_2.5_) concentrations. We assessed the performance and reliability of collocated ECMs in a subset of pregnant women participating in a randomized controlled trial in Guatemala to test a liquefied petroleum gas (LPG) cookstove (intervention) versus biomass (control) for cooking. We compared average daily (24 h) PM_2.5_ concentrations for the paired gravimetric (*n* = 219) and paired adjusted nephelometric (*n* = 221) samples using Spearman correlation and Bland–Altman agreement; we also assessed reliability based on the intraclass correlation coefficient (ICC) and root mean square error (RMSE). Median PM_2.5_ gravimetric concentrations were 93.5 *μ*g/m^3^ (interquartile range (IQR = 52.6 – 160.5) and 21.4 *μ*g/m^3^ (IQR = 12.0 – 32.0) in the control and LPG intervention groups, respectively; the median adjusted nephelometric concentrations were 83.7 *μ*g/m^3^ (IQR = 47.6 – 148 9) and 22.6 *μ*g/m^3^ (IQR = 17.5 – 29.7) in the control and LPG groups, respectively. Spearman correlations were higher in the control arm (0.91) than in the LPG intervention arm (0.67) in the gravimetric comparisons. The same trend was observed for adjusted nephelometric measurements in the control (0.92) and intervention arms (0.75). ICC values were high in both gravimetric (0.93) and nephelometric (0.95) collocations. Small differences in RMSE were observed for the gravimetric (26.69 *μ*g/m^3^) and nephelometric collocations (31.76 *μ*g/m^3^). Our findings demonstrate strong reliability between collocated ECMs for both gravimetric and adjusted nephelometric PM_2.5_ personal exposure samples in rural Guatemala.

**Trial Registration:**

ClinicalTrials.gov identifier: NCT02944682

## 1. Introduction

Household air pollution (HAP) from the combustion of biomass as fuels is the second leading cause of death among environmental and occupational risk factors around the globe. In 2021, it was associated with an estimate of 3.1 million annual deaths and 111.5 million disability-adjusted life years (DALYs) [1]. Particulate matter with an aerodynamic diameter of 2.5 *μ*m or less (PM_2.5_) is a component of HAP linked to health outcomes such as low birthweight, short gestation period, and increased risk of pulmonary and cardiovascular diseases [2–4]. Cooking with biomass is one of the main sources of HAP that occurs more frequently in low- and middle-income countries (LMICs). Generally, in LMICs, women and children are the most exposed to HAP from cooking [5].

Exposure to HAP can be modified by implementing fuel interventions [6]. As examples, the study of McCracken et al. [7] assessed exposure–response changes by implementing a biomass chimney stove intervention. In their study, personal exposure to PM_2.5_ was reduced by 38%, when comparing the chimney stove intervention against the open-fire stove. The study of Checkley et al. [8] assessed exposure–reductions and found average personal PM_2.5_ concentrations of 30 *μ*g/m^3^ on participants using an LPG stove intervention compared to 98 *μ*g/m^3^ when cooking with a biomass fuel stove. One of the most recent HAP trials, the Household Air Pollution Intervention trial (HAPIN) described by Clasen et al. [9], sought to demonstrate the effect of a randomized LPG stove and fuel intervention on health and evaluate exposure–response associations for HAP and health outcomes. In many trials and observational studies, the main method to assess HAP exposure is through surveys or questionnaires. These qualitative approaches rely on the evaluation of the fuel and stove type, which is less expensive but prone to exposure misclassification [10]. Therefore, accurate exposure assessments are needed in intervention studies to better assess potential HAP reductions.

Air pollution instruments have been developed and adapted to estimate HAP levels more precisely. Gravimetric pumps are conventional instruments for PM_2.5_ assessment, originally developed for measurements in occupational settings, which are bulky, relatively heavy, and noisy [11]. By using these pumps, aerosols are collected on filters during a specified monitoring period. The particle mass concentration can then be determined based on the weight difference of the filters before and after sampling. Size-selective inlets can be used to sample a specific size fraction of particulate matter, like PM_2.5_ [12]. Gravimetric analysis of this type is guided by the US Environmental Protection Agency’s reference method for measuring PM_2.5_ concentrations [13]. However, this method is time intensive and, as an integrated sample, does not provide temporally granular patterns of exposure [14].

The Enhanced Children’s MicroPEM (ECM), developed and patented by Research Triangle Institute (RTI) International [15], was designed to improve PM_2.5_ exposure measurements in a variety of settings. Beyond the gravimetric feature, this instrument can record concentrations with temporal resolution at user-specific intervals using a light-scattering nephelometer. The instrument also logs accelerometry to determine movement patterns, temperature, humidity, and real-time pump flow rates [16].

Few studies have evaluated the performance of the ECM relative to other instruments. Two pilot studies were conducted to test compliance and the agreement between the ECM and its previous version, the MicroPEM [15, 17]. Burrowes et al. [18] conducted an ECM validation study in Peru and assessed the correlation with a standard gravimetric measurement using a pump and a PM_2.5_ cyclone operating at 1.5 L/min (Spearman *ρ* = 0.91, 95% confidence interval (CI): 0.85, 0.97). They found a mean difference between instruments (using the Bland–Altman method) of 121.7 *μ*g/m^3^. A more recent study [19] found a statistically significant correlation between PM_2.5_ exposure in mother–children pairs using the MicroPEM and the ECM (Spearman *ρ* = 0.43, *p* < 0.001). However, they did not conduct paired (collocations) measurements in the same study subjects, but rather, they contrasted PM_2.5_ exposures as mother–children’s comparisons.

Furthermore, few studies have evaluated these instruments in duplicate as personal exposure monitors to assess the inter-unit variability. Du et al. [20] determined the correlation coefficient of 0.98 (*p* < 0.01) between duplicate ambient MicroPEMs. Burrowes et al. [18] also assessed duplicate measurements using the ECM, although the correlation and agreement results from these duplicates were not mentioned.

As noted before, there is limited knowledge about the performance of the ECM in field studies. The objective of this study was to evaluate the performance and reliability of ECMs by comparing collocated (instruments sampling at the same time, on the same subject) devices in the context of field research.

## 2. Methods

### 2.1. Study Setting.

This study is part of the HAPIN, a multi-country randomized controlled trial to test the effects of an LPG cookstove and fuel intervention in settings that rely chiefly on biomass for cooking. The study recruited pregnant women and followed them and their offspring for approximately 18 months. A description of the HAPIN trial is described by Clasen et al. [9], and the methods for exposure sampling by Johnson et al. [16]. The samples for this study are drawn from the trial’s Guatemala rural site in the Jalapa municipality. The average elevation is 1163 m above sea level with an average annual temperature of 18.3°C.

### 2.2. ECM Instrument and Sampling.

**Figure 1:**
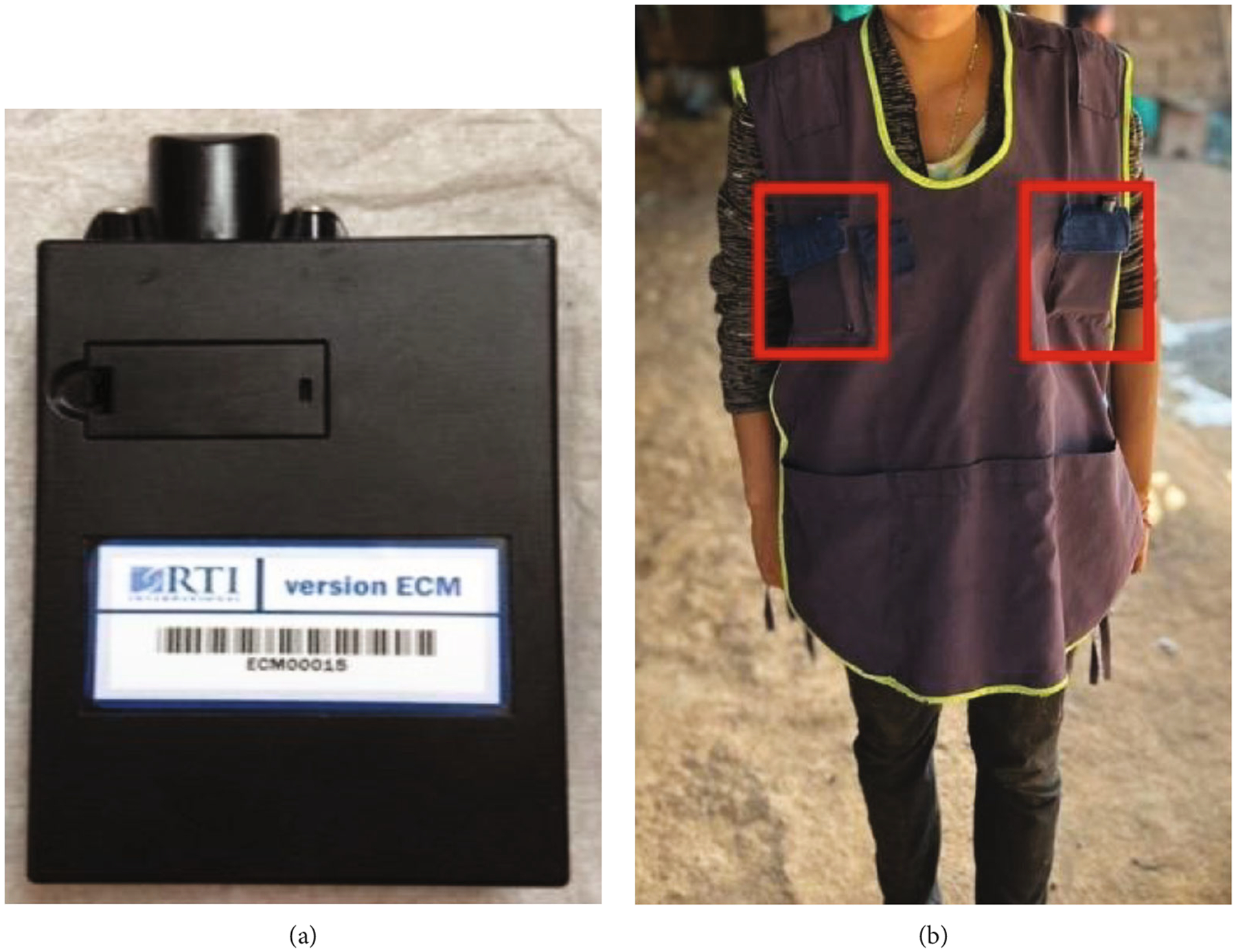
The Enhanced Children’s MicroPEM (ECM) (a) and a participant wearing an apron with two collocated ECMs, highlighted in red (b).

The ECM (Figure 1(a)) is an air monitor that weighs approximately 150 g and measures 6 × 2 × 12 cm. The instrument is powered by a lithium battery that fully charges in 4 to 6 h. We used polytetrafluoroethylene (PTFE) filters with a pore size of 2 *μ*m from Measurement Technology Laboratories (MTL; Minnesota, United States) for the gravimetric sample collection.

The MicroPEM docking station software version 2.0 was used to calibrate the nominal flow, temperature, relative humidity (RH), and nephelometer sensors, usually the day before each deployment. During the calibration process, a fitted cap was placed into the ECM’s inlet, connecting to a small HEPA-Vent filter through plastic tubing. The nominal flow was set at 0.3 L/min (±0.015 L/min) with the aid of the Gilian Gilibrator-2 flowmeter (Sensidyne; St. Petersburgh, Florida), the temperature and RH were set to the room temperature and RH displayed by an external EasyLog-21CFR-2-LCD+ (Lascar Electronics; Erie, Pennsylvania), and the nephelometer was set to 0 *μ*g/m^3^ following the MicroPEM docking station nephelometer reading. The flowmeter was also used to record the ECM’s final nominal flow shortly after each sampling ended.

Personal exposure measurements were collected for pregnant women by placing the ECMs inside front pockets of a designed apron. Participants wore the instruments for 24 h and were instructed not to take off the equipment unless the activity was impeded by or damage could be sustained to the device, such as bathing or sleeping. Study data were collected and managed using Research Electronic Data Capture (REDCap) electronic data capture tools hosted at Emory University (Atlanta, Georgia, United States) [21, 22]. REDCap is a secure, web-based software platform designed to support data capture for research studies. We collocated two ECMs in a subset of 112 randomly selected participants from the HAPIN trial in Guatemala (Figure 1(b)) and operated them over the same 24-h periods. For each participant, the samples were collected for at least three of the six total time-point–specified exposure pregnancy visits from the HAPIN study. More details about personal exposure measurements can be found in Johnson et al. [16].

### 2.3. Gravimetric and Nephelometric Data and Processing.

Filters were weighed before and after sampling on a 1-*μ*g-resolution microbalance (Sartorius Cubis MSA6.6S-000-DF) in the gravimetric laboratory at the Environmental Health Sciences Department at the University of Georgia (Athens, Georgia, United States) according to the 2016 US Environmental Protection Agency’s protocol [13].

Mass deposition (*M*_2.5_) was calculated as the average weight difference between two pre- and two post–weight measurements for each filter, in micrograms. Sample volume (*V*) in cubic meters was estimated with the following equation:

$$V=Q_{\text{avg}}\times T\times 10^{-3}$$

where *Q*_avg_ is the average flow rate in liters per minute and *T* is the sample duration in minutes.

The mass deposition was corrected by subtracting the median of blank field filters. The 24-h average PM_2.5_ mass concentrations were estimated using the following equation:

$${\text{PM}}_{2.5}=\frac{M_{2.5}}{V}$$

Nephelometric data was calculated with the real-time data from the ECM by first baseline adjusting the first percentile of the data to the nephelometric limit of detection (LoD) of 10 *μ*g/m^3^, representative of a typical relatively clean ambient concentration. Then, the 1-min average data was compiled into 24-h averages. Finally, we applied a linear regression model using log_10_ nephelometric and the valid gravimetric sample averages to the baseline-adjusted nephelometric 24-h averages, producing an adjusted nephelometric value.

### 2.4. Quality Assurance and Quality Control.

We only included valid gravimetric data points after following the quality assurance/quality control (QA/QC) three-stage process from the HAPIN trial [23]. This involved (1) valid pre- and post–flow measurements of the ECMs, (2) removal of damaged filters, and (3) valid instrument duration (24 ± 4 h), flow (0.3 ± 0.1 L/min), and inlet pressure (95th percentile < 5 in. H_2_O). The quality control criteria for nephelometric data included only duration and inlet pressure from the real-time data points. The LoD was estimated as three times the standard deviation of blank filters’ mass deposition. The values below the LoD were replaced with LoD/2^0.5^.

### 2.5. Statistical Analyses.

We conducted two main comparisons to assess the ECM reliability: gravimetric against gravimetric and nephelometric against nephelometric collocations (pairs). Spearman correlation coefficients and their 95% CIs were estimated to determine the strength of the comparisons. The Bland–Altman method was used as a measure of agreement for each of the comparisons. This method consisted in plotting the averages against the absolute differences of the paired concentrations, so then, the mean differences are estimated [24]. The limits of agreement were determined based on the 95% CIs of the mean differences and standard deviations. The reliability for each ECM comparison was estimated with the natural log–transformed concentrations using the intraclass correlation coefficient (ICC) one-way random effects model, described as

$$\frac{{\text{MS}}_{\text{R}}-{\text{MS}}_{\text{W}}}{{\text{MS}}_{\text{R}}}$$

where MS_R_ is the mean square of observations and MS_W_ is the mean square for residual sources of variances.

The ICC is one of the recommended methods to assess reliability between measurements since it reflects not only the degree of correlation but also the agreement [25–27]. The root mean square error (RMSE) was computed as an additional method to assess the reliability of the comparisons.

Spearman correlation, Bland–Altman agreement, ICC, and RMSE were estimated after stratifying the ECM comparisons by groups derived from the study arms. The control comparisons include all control group (postrandomization) samples and baseline samples (intervention and control prerandomization); given in theory, these represent only the use of biomass stoves for cooking. The intervention comparisons include all intervention samples after randomization. Correlations and agreement were also assessed by splitting the data into ranges based on the 25th, 50th, and 75th percentiles to further evaluate the effect of concentration ranges. The concentrations were then natural log transformed to conduct parametric testing. The one-way analysis of variance (ANOVA) was computed to test for statistically significant differences in the concentration between both intervention arms. All statistical analyses were performed in R version 4.2.

## 3. Results

### 3.1. Study Population.

Among the 112 participants included in this study, 50 corresponded to the intervention and 62 to the control arm. A detailed description of demographic and maternal characteristics at enrollment of the study population, categorized by study arm, is presented in the supporting tables (Table S1). After applying the QA/QC three-stage process from HAPIN, a total of 228 pregnancy visits were included in the analysis. A more detailed description can be found in Table S2 and Table S3.

### 3.2. PM_2.5_ Concentrations.

**Figure 2:**
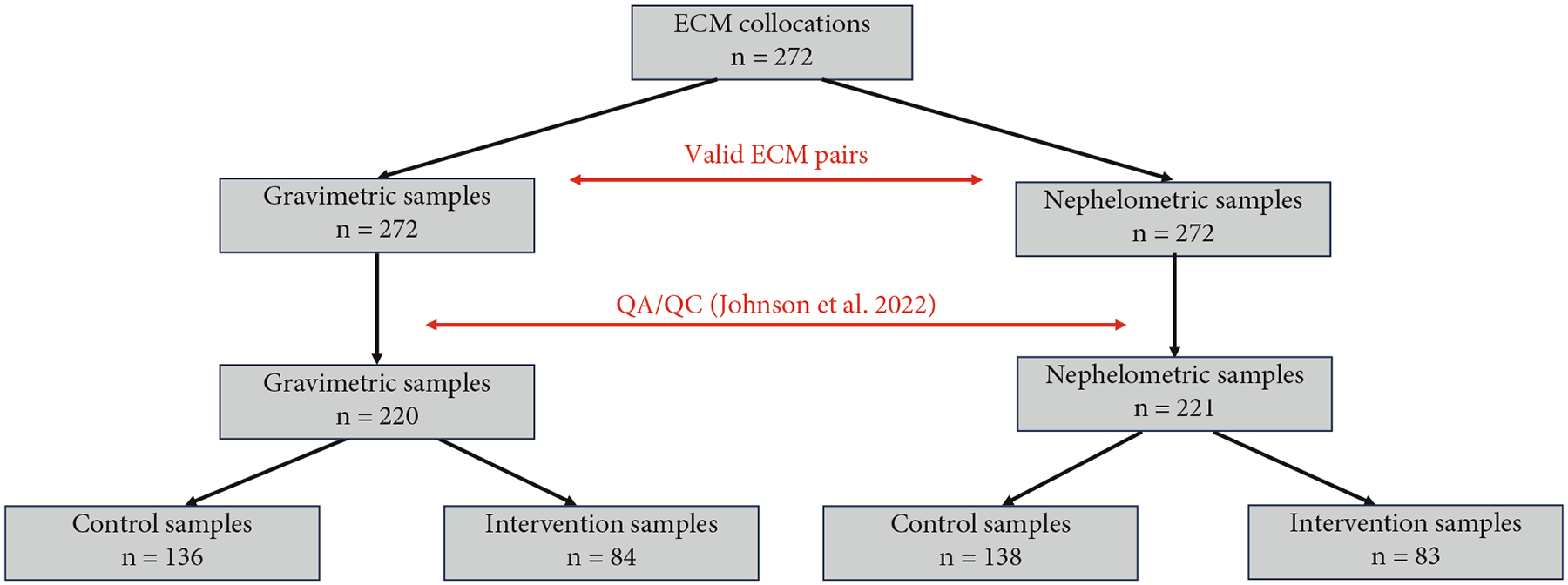
Flowchart for the selection of collocations in the gravimetric and nephelometric samples. *n* = collocations and “valid ECM pairs” is the number of valid samples from both collocated ECMs. Control samples include all control pairs (postrandomization) and all pairs from baseline (control and intervention, prerandomization). The intervention samples include only all postrandomization intervention pairs.

In total, 220 gravimetric collocations were available after applying the QA/QC three-stage process. Of these, 136 collocations (61.8%) were from the control group (control and intervention, prerandomization) and 84 (38.2%) to the intervention group. Also, 221 nephelometric collocations were available, 138 (62.4%) from control and 83 (37.6%) from the intervention group (Figure 2).

**Figure 3:**
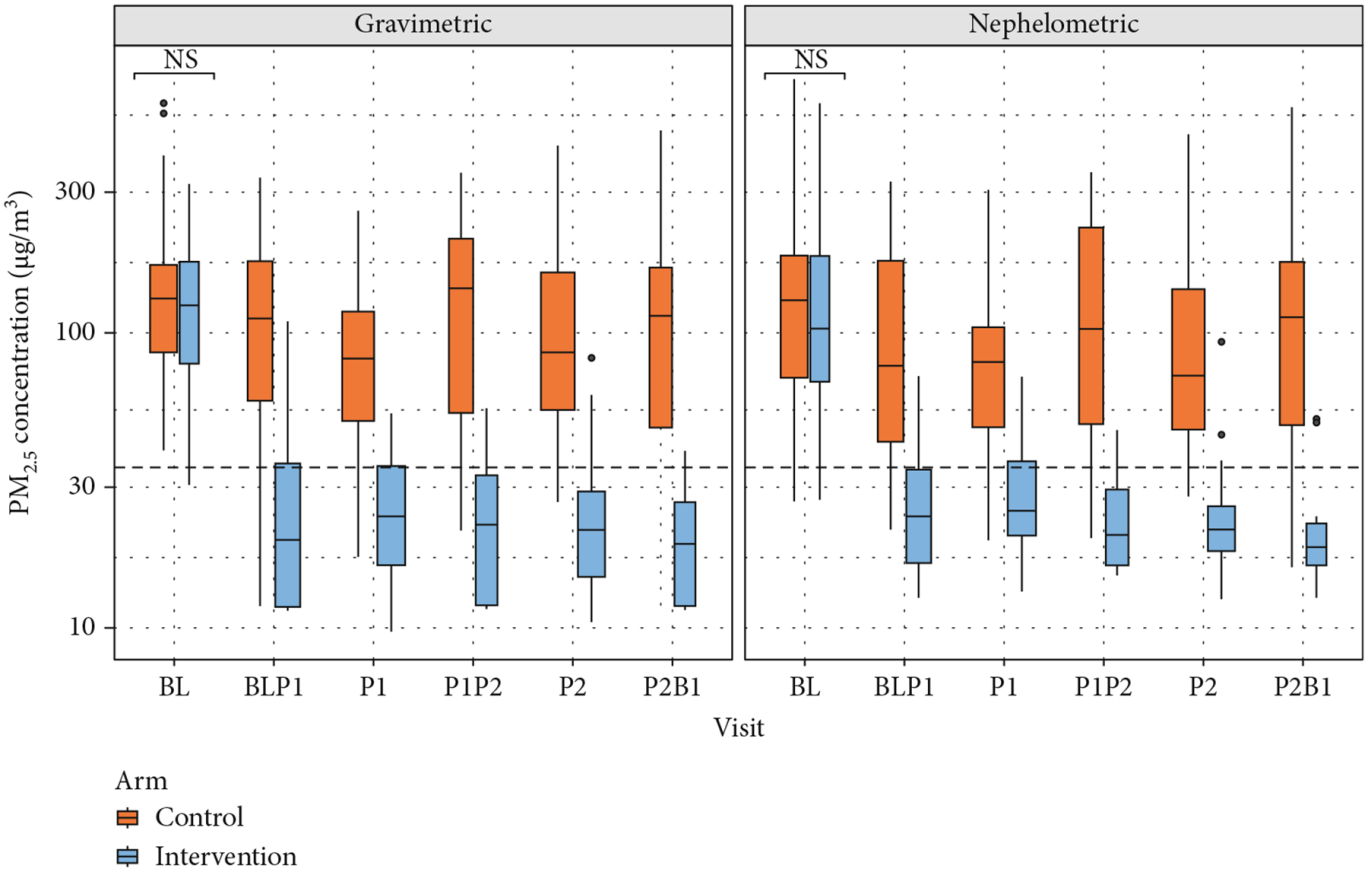
Boxplots of PM_2.5_ concentrations for each visit by type of measurement. Nonsignificant differences between study arms are shown as “NS.” The dashed black line represents the World Health’s Organization (WHO) 24-h average air quality guideline of PM_2.5_ = 15 *μ*g/m^3^. “*n*” denotes the sample size.

There were 440 and 442 individual gravimetric and adjusted nephelometric 24-h average PM_2.5_ concentrations, respectively, available for analysis. The concentrations by type of visit and study arm are summarized in Tables S4 and S5 and in Figure 3. PM_2.5_ postrandomization concentrations were lower in the intervention group compared to the control group for both gravimetric (median (IQR) micrograms per cubic meter = 21.4 (12.0–32.0) vs. 93.5 (52.6–160.5)) and nephelometric median (IQR) micrograms per cubic meter = 22.6 (17.5–29.7) vs. 83.7 (47.6–148.9)) samples. Based on the analysis of variance, there was a statistically significant difference in the 24-h average PM_2.5_ concentrations between the control and intervention groups (*p* < 0.001) for both gravimetric and nephelometric comparisons.

### 3.3. Evaluation of the Gravimetric and Nephelometric Comparisons.

**Figure 4:**
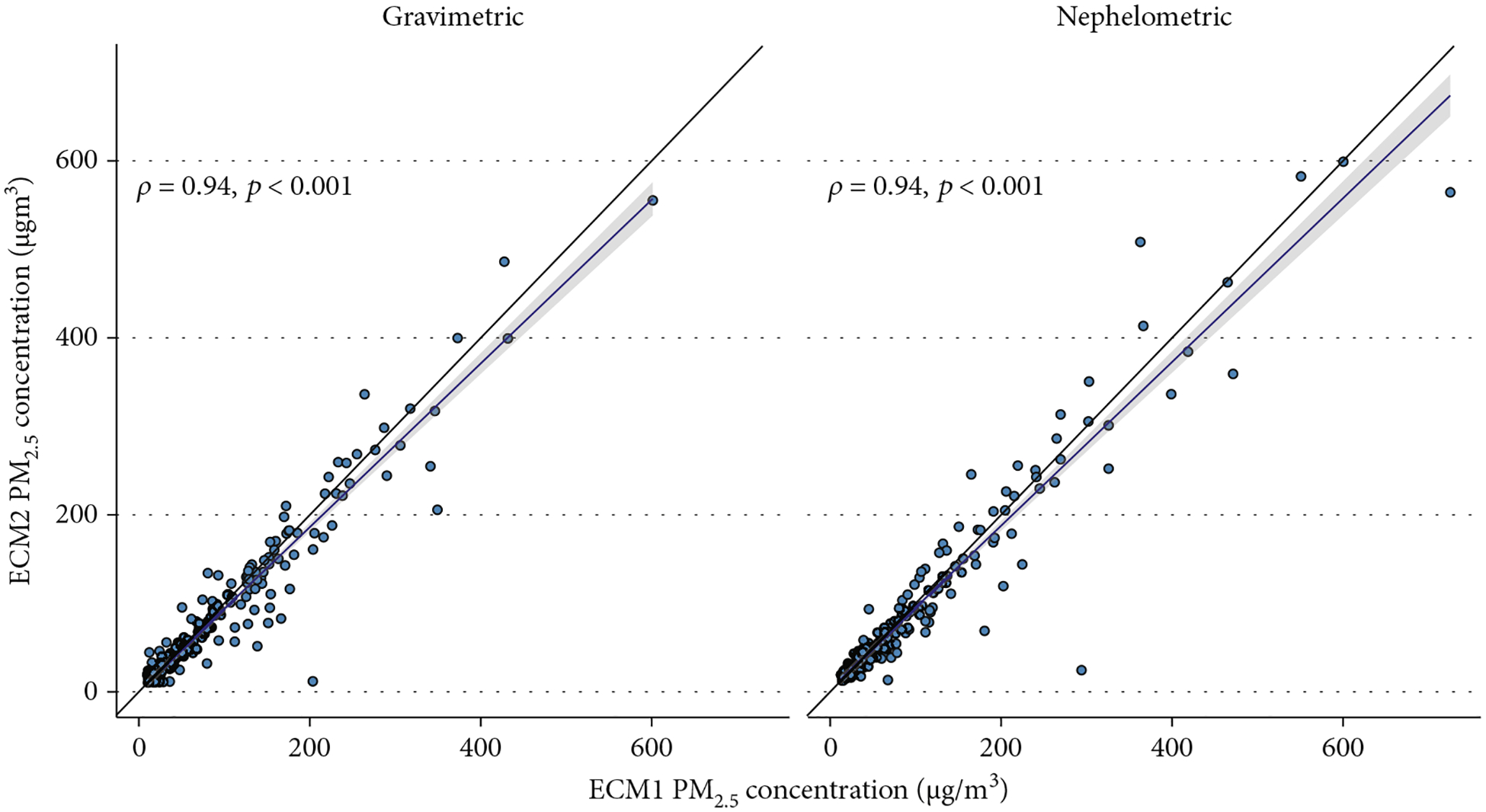
Correlation plots for the ECM gravimetric and nephelometric comparisons.

**Figure 5:**
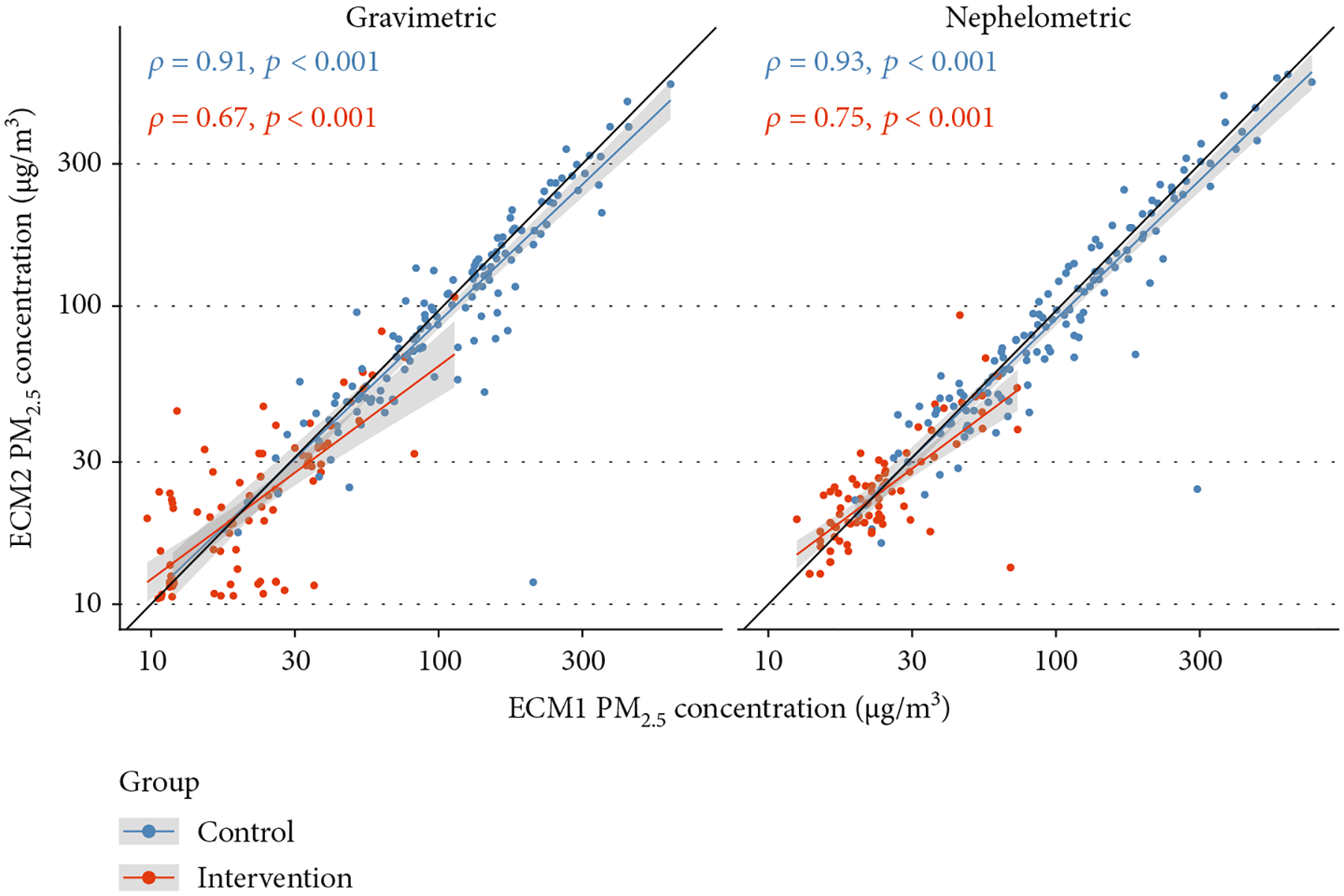
Correlation plots for the ECM control and intervention group comparisons.

**Figure 6:**
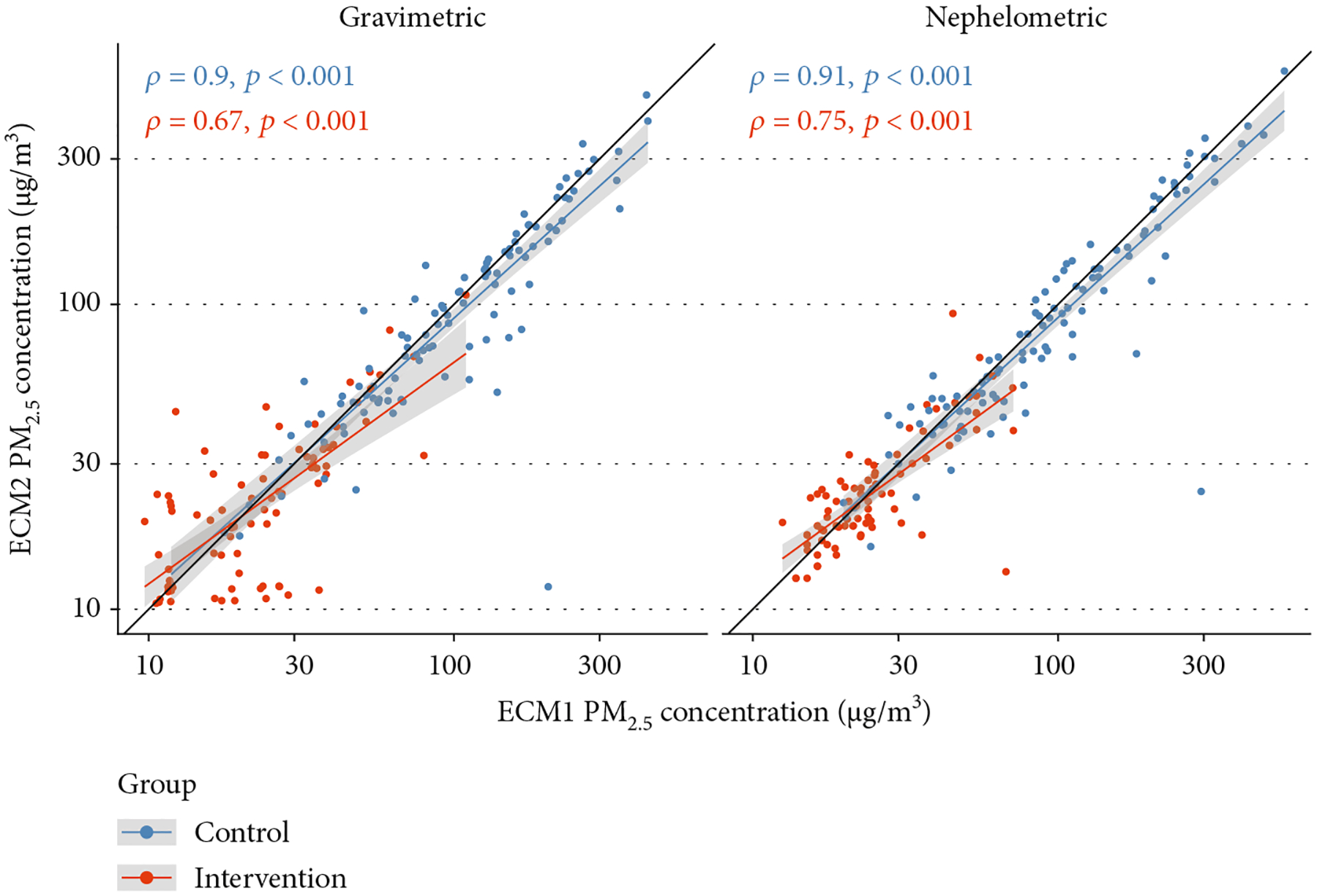
Correlation plots for the ECM control and intervention group comparisons, only with postrandomization samples.

Overall, correlations were high for both gravimetric and adjusted nephelometric comparisons (*ρ* = 0.94) Figure 4. When stratifying by study group, correlations differed between both groups. The correlations were lower in the intervention group when compared to the control group, for both gravimetric and nephelometric comparisons (Table 1 and Figure 5). An additional correlation analysis which includes only samples postrandomization (all samples except baseline) shows that the correlation pattern is similar to the observed using all the samples (Figure 6).

**Figure 7:**
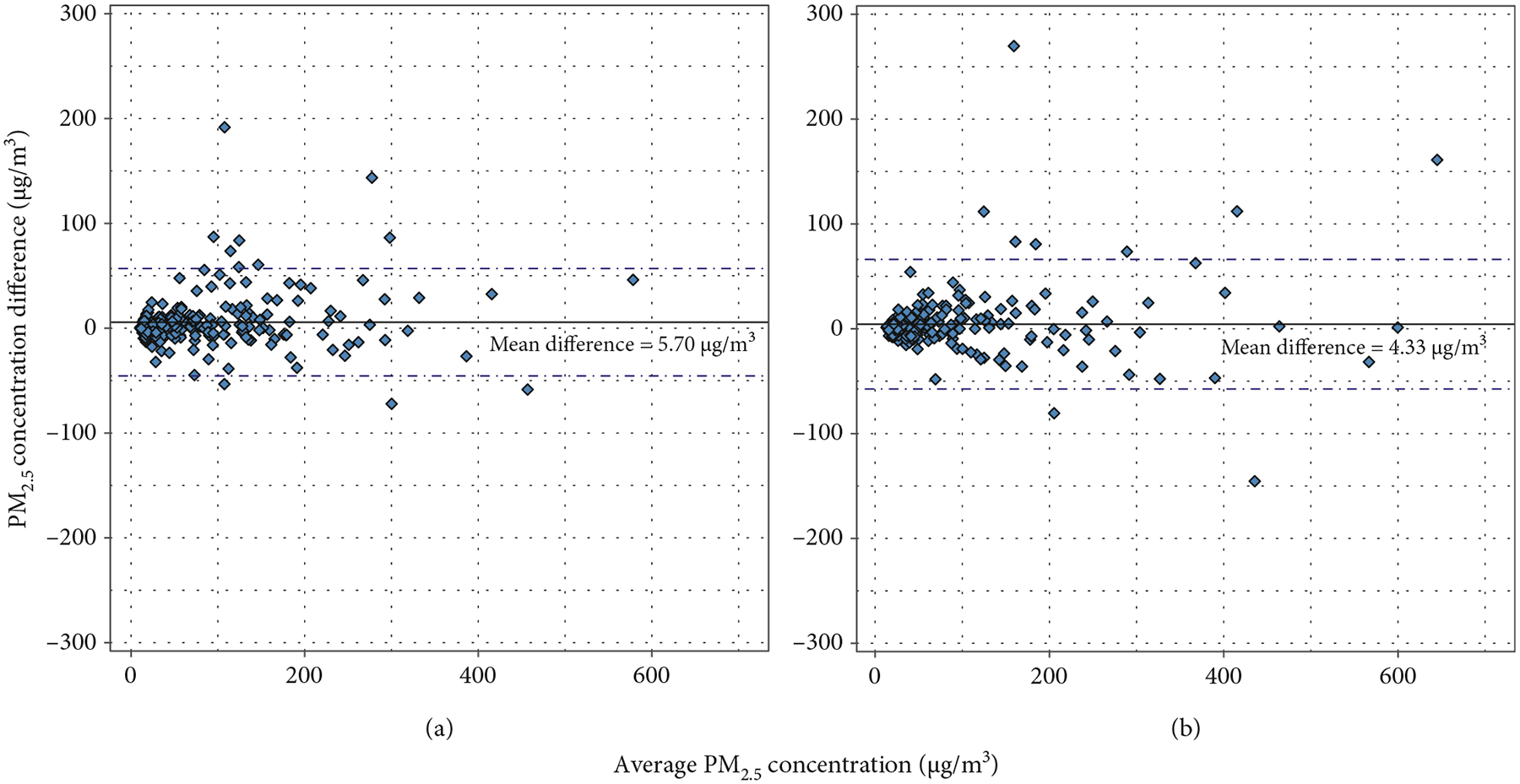
Bland–Altman agreement plots for the ECM gravimetric and nephelometric measurements. (a) The gravimetric results and (b) nephelometric results.

**Figure 8:**
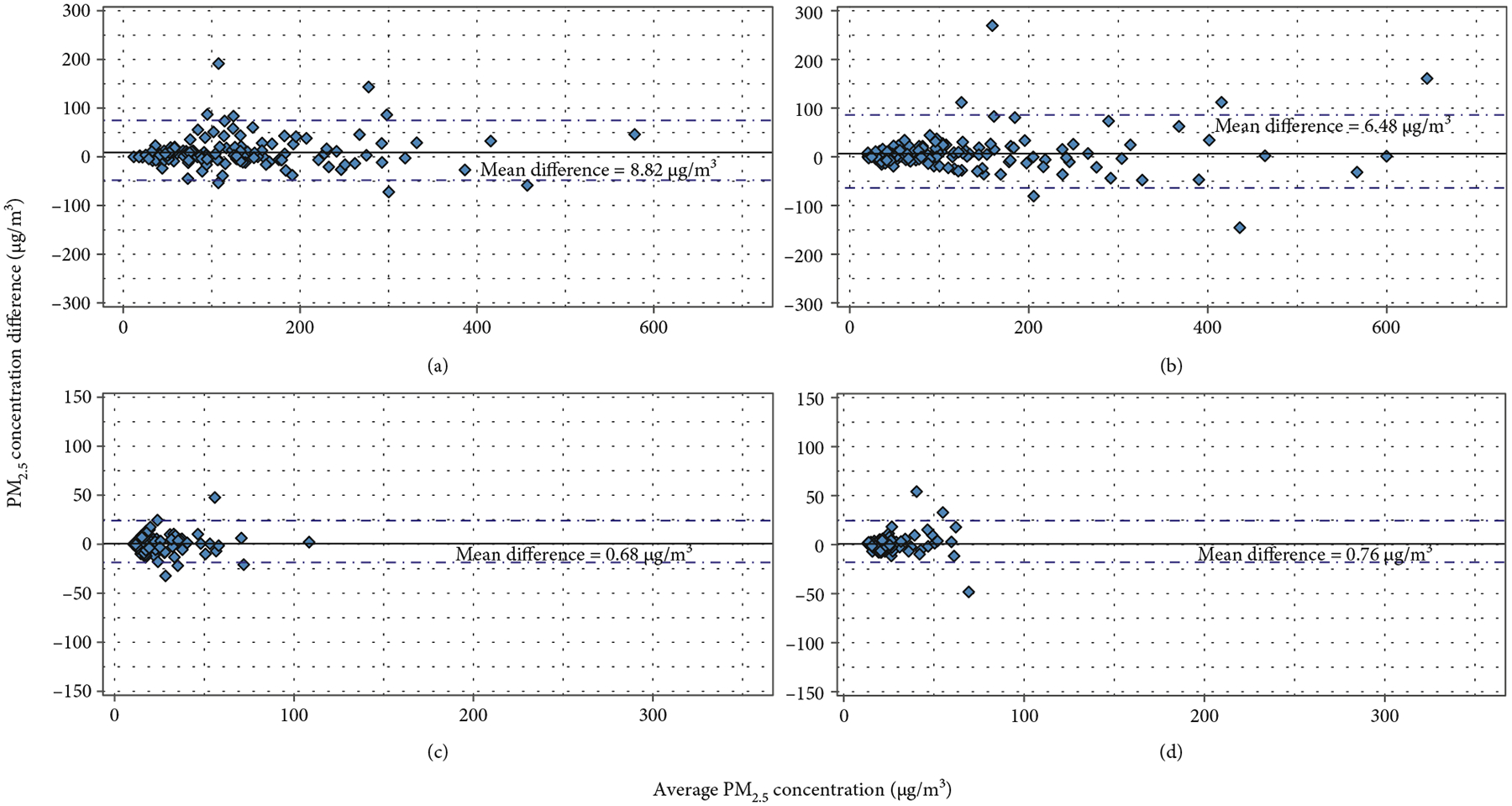
Bland–Altman agreement plots. Top: control group, where (a) shows the gravimetric results and (b) shows the nephelometric results. Bottom: intervention group, where (c) shows the gravimetric results and (d) shows the nephelometric results.

Overall, there was strong agreement in the gravimetric and nephelometric ECM comparisons (Figure 7). The agreement was similar in the gravimetric (5.70 *μ*g/m^3^) and nephelometric comparisons (4.33 *μ*g/m^3^). When stratifying by study group in both gravimetric and nephelometric collocations (Figure 8), a higher agreement (meaning a lower mean difference) was observed in the intervention group compared to the control group.

When observing the data split by percentiles, a similar pattern in the agreement was observed in both gravimetric and nephelometric comparisons (Table 2 and Figures S1–S4). The data below the 25th and 50th percentiles showed higher agreement values in both gravimetric and nephelometric comparisons, compared to the data above the 50th and 75th percentiles. Meanwhile, the correlations were higher in the above the 50th and 75th percentiles compared to the lower than 50th and 25th percentiles (Table 2 and Figure S5).

### 3.4. Reliability.

ICC values were 0.93 and 0.95 for the gravimetric and adjusted nephelometric comparisons, respectively (Table 1). When stratifying by study group, ICC values were 0.89 in gravimetric comparisons and 0.93 for nephelometric comparisons in the control group. In contrast, the reliability was 0.72 in gravimetric and 0.76 in nephelometric comparisons in the intervention group. The overall RMSE was also observed to be lower in the gravimetric collocations (26.69 *μ*g/m^3^) compared to the nephelometric collocations (31.76 *μ*g/m^3^). In the control group comparisons, the RMSE was lower in the gravimetric pairs compared to the nephelometric pairs, but in the intervention group, they were similar when contrasting gravimetric and nephelometric pairs (Table 1).

## 4. Discussion

Collocated gravimetric and adjusted nephelometric measurements were highly correlated and demonstrated high agreement. We found high reliability between collocated ECMs from PM_2.5_ personal exposure measurements in Jalapa, Guatemala, as seen by the ICC values in the gravimetric (0.93) and adjusted nephelometric (0.95) comparisons.

The ECM is a relatively new instrument developed in 2015 by RTI International, and since its conception, it has been used for field research and indirectly as a validation point, but not like the evaluation presented in this study. The studies by Johnson et al. [23] and Liao et al. [28] highlight the use of the ECM for PM_2.5_ personal exposure measurements, and other studies [29–33] highlight the applicability of these measurements for exposure–response or intention-to-treat analysis on various health outcomes. However, it was the ECM validation analyses by Chartier [17] and Burrowes et al. [18] that justified the use of this instrument in the mentioned studies. First, it is important to describe the most formidable features of the ECM to consider before using it in field campaigns. The ECM is a small instrument and relatively silent during its operation. The battery proved to be long lasting (more than 24 h), and it was observed that it completely charges in less than 4 h. Limitations of this device include the difficulty in handling the 15-mm filter and the device during calibration. These issues are resolved through repeated training of the staff in charge of this task, which also includes conducting maintenance procedures to avoid the possibility of faulty units. However, the most attractive feature is its capability to conduct both real-time measurements, through the nephelometer sensor, and collection of gravimetric samples.

Gravimetry has been considered a “gold standard” for PM_2.5_ exposure assessment [34, 35], but our results show that the adjusted nephelometric concentrations can be used in cases where the sample filter is damaged, and the gravimetric concentration cannot be easily estimated. However, this does not imply that we should rely on the nephelometric concentrations only, since it was shown that the nephelometric data had to be adjusted based on the average of a group of valid gravimetric samples and a log_10_ linear regression model derived from these. Another reason to not rely solely on nephelometric concentrations is that filter samples can be further analyzed (e.g., via source apportionment methods) to derive additional chemical or physical information on the samples.

Mathematically speaking, the Spearman method is not an indicator of reliability since it only evaluates correlation of paired samples [27]; however, it served as a starting point to assess concordance. The Spearman correlations were different when comparing the measurements between study groups and by data percentiles (Figure 5 and Table 2). The additional correlation analysis, which removed all baseline (preintervention samples) comparisons, demonstrated that reducing the sample size of the comparisons did not produce a significant effect on the correlation strength (Figure 6). The previous analyses support that the different *ρ* values can be explained due to the differences in concentrations.

The ranges, means, and medians are significantly higher in control participants compared to the intervention. This could suggest that the ECM sensor is less exact on estimating PM_2.5_ for lower concentrations, driven by instrument/analytical error in concentrations closer to the LoD. The reduced range of exposures in the intervention group comparisons also explains the lower correlation, which is related to being closer to the measurement error range of single observations. This was further confirmed by the correlations when the data was analyzed by percentiles. However, a high agreement in the intervention group samples and in the lowest percentiles was observed, but it remains to be evaluated if this agreement is absolute or relative when moving from lower to higher concentrations. To summarize the previous, in the context of field research, this suggests that when using the ECM to measure PM_2.5_, there will likely be smaller correlations among duplicates at exposure levels below the air quality guidelines with the tradeoff of a higher absolute agreement.

The correlations and ICC values produced similar results, showing stronger associations in the control group, while weaker associations in the intervention group. When comparing the ICCs in gravimetric samples and nephelometric values, the absolute difference was the same if stratified by control and intervention groups (ICC difference = 0.04). Previous studies have shown that PM concentrations estimated with nephelometer sensors are sensitive to variations in shape, size, and density of the particles [36, 37] compared to relying on mass deposition as an estimate of concentrations. However, for this study, it was not the case when conducting gravimetric and nephelometric comparisons.

If we want to observe how these comparisons are representative of the HAPIN Guatemala study population, Table S1 shows that the proportions of some of the household and maternal characteristics of this subset are similar to the ones reported by Johnson et al. [23]. Likewise, although the scope of this study was not to report the personal PM_2.5_ exposure reductions, we observed that this is consistent with findings from the pilot study from HAPIN [28], the prebirth exposures reported by Johnson et al. [23], and similar to exposure reductions reported from other LPG intervention trials [38, 39]. This suggests that the randomized process that assigned participants with ECM collocations was effective for accounting to a representative subsample of the HAPIN trial in Guatemala.

One additional analysis that could complement our findings about the real-time sensor is to do a moving average analysis and visual inspection of the raw nephelometric concentrations. In addition, three potential sampling limitations are recognized from this study. First is the lack of comparisons of the ECM with another PM_2.5_ instrument. As seen in previous studies [18, 20, 40, 41], PM_2.5_ instruments have been compared to a newer or different model to assess their performance. Second, the only collocations were conducted for personal measurements in pregnant women participants and not in other participants or in household areas. And third, this study includes data from only one of the countries that were part of HAPIN. In summary, including other collocations would further contribute to the generalizability of these findings. Despite these limitations, the current study demonstrates the strong performance if the ECM in Guatemala, as an instrument to measure PM_2.5_ in a large, randomized intervention trial.

## 5. Conclusions

There was high reliability between collocated ECMs from PM_2.5_ personal exposure measurements in Jalapa, Guatemala, as seen by the ICC values in the gravimetric (0.93) and adjusted nephelometric (0.95) comparisons. Collocated gravimetric and adjusted nephelometric measurements showed high agreement and demonstrated to be highly correlated. The ranges in PM_2.5_ concentrations were different between biomass and LPG intervention groups, which is reflected in the reliability and correlations by comparing both groups. Overall, the ECM showed strong performance to measure PM_2.5_ in rural Guatemala.

## Supplementary Material

Mollinedo_IndoorAir_2025_SI

Additional supporting information can be found online in the Supporting Information section. *(Supporting Information)* Table S1: Demographic and maternal characteristics of the study population at enrollment. Table S2: Total visits categorized by time-point–specified exposure pregnancy visit. Table S3: Number of participants categorized by total number of visits. Table S4: Statistical summary of the individual gravimetric PM_2.5_ concentrations. Table S5: Statistical summary of the individual nephelometric PM_2.5_ concentrations. Figure S1: Bland–Altman agreement plots for the ECM gravimetric and nephelometric comparisons at concentrations below the 25th percentile. Figure S2: Bland–Altman agreement plots for the ECM gravimetric and nephelometric comparisons at concentrations between the 25th and 50th percentiles. Figure S3: Bland–Altman agreement plots for the ECM gravimetric and nephelometric comparisons at concentrations between the 50th and 75th percentiles. Figure S4: Bland–Altman agreement plots for the ECM gravimetric and nephelometric comparisons at concentrations above the 75th percentile. Figure S5: Correlation plots for the ECM gravimetric and nephelometric comparisons by data percentiles.

## Figures and Tables

**Table 1:** Summary of correlations, intraclass coefficients (ICC), and root mean square error (RMSE) by type of comparison and by study group. Control samples include all control pairs (postrandomization) and all pairs from baseline (control and intervention, prerandomization), and the intervention samples include only all postrandomization intervention pairs.

Comparison	Group	*N*	Spearman correlation (95% CI)	ICC (95% CI)	RMSE (*μ*g/m ^3^)
Gravimetric	Overall	220	0.94 (0.89–0.96)	0.93 (0.91–0.95)	26.69
	Control	136	0.91 (0.84–0.96)	0.89 (0.86–0.92)	32.98
	Intervention	84	0.67 (0.51–0.80)	0.72 (0.61–0.81)	10.22
Nephelometric	Overall	221	0.94 (0.90–0.97)	0.95 (0.94–0.96)	31.76
	Control	138	0.92 (0.85–0.97)	0.93 (0.90–0.95)	39.38
	Intervention	83	0.75 (0.58–0.87)	0.76 (0.66–0.84)	10.34

**Table 2:** Summary of correlations and agreement by type of comparison and by percentiles.

Percentile	Agreement		Correlations	
	Gravimetric	Nephelometric	Gravimetric	Nephelometric
< 25th	1.95	0.75	0.31	0.54
25th-50th	1.80	0.25	0.72	0.67
50th-75th	3.84	6.86	0.73	0.77
> 75th	17.48	12.57	0.86	0.82

## Data Availability

The data that support the findings in this study will be available upon request.
