# Supplementary material for: Comparing Performance and Reliability of Collocated Enhanced Children’s MicroPEM (ECM) on Gravimetric and Nephelometric PM2.5 Personal Exposure Samples in Field Measurements in Rural Guatemala
Source: Indoor Air. Author manuscript; Available in PMC 2026 May 1. (PMC13132538; doi:10.1155/ina/8812602)
Supplement: Mollinedo_IndoorAir_2025_SI [file NIHMS2165633-supplement-Mollinedo_IndoorAir_2025_SI.docx]

**Supporting Information:**

**“Comparing Performance and Reliability of Collocated Enhanced Children’s MicroPEM (ECM) on Gravimetric and Nephelometric PM_2.5_ Personal Exposure Samples in Field Measurements in Rural Guatemala”**

Table S1: Demographic and maternal characteristics of the study population at enrollment. (Intervention and Control groups represent the actual group the participants were allocated as part of the HAPIN trial)

| **Characteristics at enrollment** | **Total** | **Intervention** | **Control** |
| --- | --- | --- | --- |
|  | **N= 112** | **N= 50 (44.6%)** | **N= 62 (55.4%)** |
| **Participant’s age (years)** |  |  |  |
| Mean ± SD | 24.7 ± 4.61 | 24.7 ± 4.62 | 24.8 ± 4.65 |
| 18-23 | 57 (50.9%) | 25 (50%) | 32 (51.6%) |
| 24-29 | 36 (32.1%) | 16 (32%) | 20 (32.3%) |
| 30-35 | 19 (17%) | 9 (18%) | 10 (16.1%) |
| **Gestational age (weeks)** |  |  |  |
| Mean ± SD | 13.8 ± 2.76 | 13.9 ± 2.43 | 13.7 ± 3.0 |
| 9-12 | 56 (50%) | 25 (50%) | 31 (50%) |
| 13-16 | 39 (34.8%) | 19 (38%) | 20 (32.3%) |
| 17-20 | 17 (15.2%) | 6 (12%) | 11 (17.7%) |
| **Number of biomass stoves** |  |  |  |
| One | 39 (34.8%) | 21 (42%) | 18 (29%) |
| Two | 68 (60.7%) | 27 (54%) | 41 (66.2%) |
| Three or more | 5 (4.5%) | 2 (4%) | 3 (4.8%) |
| **Main stove type** |  |  |  |
| Open fire | 87 (77.7%) | 39 (78%) | 48 (77.4%) |
| Chimney | 25 (22.3%) | 11 (22%) | 14 (22.6%) |
| **Trash disposal** |  |  |  |
| Burn it | 21 (18.8%) | 8 (16%) | 13 (21%) |
| Other* | 91 (81.2%) | 42 (84%) | 49 (79%) |
| **Lighting source** |  |  |  |
| Electricity | 97 (86.6%) | 43 (86%) | 54 (87.1%) |
| Fire source** | 11 (9.8%) | 5 (10%) | 6 (9.7%) |
| Other^ǂ^ | 4 (3.6%) | 2 (4%) | 2 (3.2%) |
| **Tobacco smoking in home** |  |  |  |
| Yes | 7 (6.2%) | 4 (8%) | 3 (4.8%) |
| No | 105 (93.8%) | 46 (92%) | 59 (95.2%) |
| **Education level** |  |  |  |
| None | 7 (6.3%) | 3 (6%) | 4 (6.5%) |
| Primary | 77 (68.7%) | 34 (68%) | 43 (69.4%) |
| Secondary | 23 (20.5%) | 11 (22%) | 12 (19.3%) |
| Vocational | 4 (3.6%) | 1 (2%) | 3 (4.8%) |
| College or University | 1 (0.9%) | 1 (2%) | --- |
| **Occupation** |  |  |  |
| Household work | 103 (92%) | 44 (88%) | 59 (95.2%) |
| Other^#^ | 9 (8%) | 6 (12%) | 3 (4.8%) |
| **Family size** |  |  |  |
| Mean ± SD | 4.8 ± 2.28 | 4.9 ± 2.08 | 4.8 ± 2.44 |
| 2 – 4 | 60 (53.6%) | 26 (52%) | 34 (54.8%) |
| 5 – 7 | 37 (33%) | 18 (36%) | 19 (30.7%) |
| 8 – 11 | 15 (13.4%) | 6 (12%) | 9 (14.5%) |
| **Primary cook** |  |  |  |
| Pregnant participant | 96 (85.7%) | 44 (88%) | 52 (83.9%) |
| Mother/Mother-in-law | 15 (13.4%) | 6 (12%) | 9 (14.5%) |
| Sister/Sister-in-law | 1 (0.9%) | --- | 1 (1.6%) |

*Trash buried, thrown away, collected by the government, compost and fed to animals

**Candles, traditional stove and oil lamps

^ǂ^Solar light, battery torches and solar panel

^#^Housemaid, teaching, agriculture, commercial or industry job

Table S2: Total visits categorized by time-point specified exposure pregnancy visit. (BL= Baseline or <20 weeks of gestation; BL-P1: Assessment intermediate between BL and P1; P1: Assessment between 24-28 weeks of gestation; P1-P2: Intermediate between P1 and P2; P2: 32-36 weeks of gestation, P2-B1: One month after P2 or approximately one week before birth)

| **Visit** | **Number** | **Percentage** |
| --- | --- | --- |
| **BL** | 31 | 14% |
| **BLP1** | 35 | 15% |
| **P1** | 52 | 23% |
| **P1P2** | 31 | 14% |
| **P2** | 51 | 22% |
| **P2B1** | 28 | 12% |
| **Total** | **228** | **100%** |

Table S3: Number of participants categorized by total number of visits.

| **Number of visits** | **Number of Participants** | **Percentage** |
| --- | --- | --- |
| **1** | 35 | 31% |
| **2** | 40 | 36% |
| **3** | 36 | 32% |
| **5** | 1 | 1% |
| **Total** | **112** | **100%** |

Table S4: Statistical summary of the individual gravimetric PM_2.5_ concentrations.

| **Statistic** | **Baseline** | | **Baseline-P1** | | **P1** | | **P1-P2** | | **P2** | | **P2-B1** | |
| --- | --- | --- | --- | --- | --- | --- | --- | --- | --- | --- | --- | --- |
|  | **Control** | **Intervention** | **Control** | **Intervention** | **Control** | **Intervention** | **Control** | **Intervention** | **Control** | **Intervention** | **Control** | **Intervention** |
| **N Samples** | 40 | 20 | 34 | 34 | 56 | 42 | 32 | 26 | 62 | 40 | 28 | 24 |
| **Mean**  **(SD)** | 163.9  (128.9) | 140.6  (86.2) | 121.0  (75.3) | 31.9  (27.3) | 96.3  (61.4) | 26.1  (12.9) | 143.0  (96.3) | 25.0  (13.8) | 119.8  (89.3) | 23.8  (14.0) | 137.9  (120.2) | 20.3  (8.5) |
| **Range** | 40.0 – 601.4 | 30.5 – 320.0 | 11.8 – 336.3 | 11.4 – 109.5 | 17.4 – 259.5 | 9.7 – 53.4 | 21.4 – 349.3 | 11.6 – 55.5 | 26.7 – 431.8 | 10.5 – 82.3 | 11.8 – 486.1 | 11.5 – 39.8 |
| **Median**  **(IQR)** | 130.8  (85.9 – 170.1) | 124.0  (78.6 – 174.4) | 112.8  (59.0 – 175.1) | 19.9  (11.8 – 36.1) | 81.9  (50.3 – 118.1) | 23.9  (16.3 – 35.4) | 141.8  (53.5 – 208.7) | 22.4  (11.9 – 33.9) | 85.9  (54.8 – 160.3) | 21.5  (14.9 – 29.0) | 114.3  (47.7 – 166.7) | 19.3  (11.9 – 26.7) |

Table S5: Statistical summary of the individual nephelometric PM_2.5_ concentrations.

| **Statistic** | **Baseline** | | **Baseline-P1** | | **P1** | | **P1-P2** | | **P2** | | **P2-B1** | |
| --- | --- | --- | --- | --- | --- | --- | --- | --- | --- | --- | --- | --- |
|  | **Control** | **Intervention** | **Control** | **Intervention** | **Control** | **Intervention** | **Control** | **Intervention** | **Control** | **Intervention** | **Control** | **Intervention** |
| **N Samples** | 40 | 20 | 36 | 34 | 56 | 44 | 32 | 28 | 58 | 40 | 32 | 22 |
| **Mean**  **(SD)** | 168.6  (156.0) | 182.1  (185.0) | 112.0  (91.7) | 29.0  (16.9) | 93.8  (70.0) | 30.0  (14.1) | 137.7  (101.9) | 24.4  (10.1) | 119.1  (103.2) | 24.7  (12.9) | 148.9  (141.4) | 21.5  (9.9) |
| **Range** | 26.8 – 725.5 | 27.2 – 600.3 | 21.5 – 325.8 | 12.6 – 71.4 | 19.8 – 305.6 | 13.3 – 71.1 | 20.2 – 350.6 | 15.1 – 46.9 | 27.9 – 471.3 | 12.5 – 93.3 | 16.0 – 582.5 | 12.6 – 51.1 |
| **Median**  **(IQR)** | 129.1  (70.4 – 182.9) | 104.2  (68.4 – 185.2) | 77.4  (42.8 – 175.6) | 23.8  (16.6 – 34.5) | 79.7  (47.9 – 104.5) | 24.9  (20.6 – 36.7) | 103.4  (49.1 – 227.7) | 20.7  (16.3 – 29.4) | 71.8  (47.0 – 140.7) | 21.6  (18.2 – 25.9) | 113.0  (48.7 – 174.3) | 18.8  (16.3 – 22.6) |


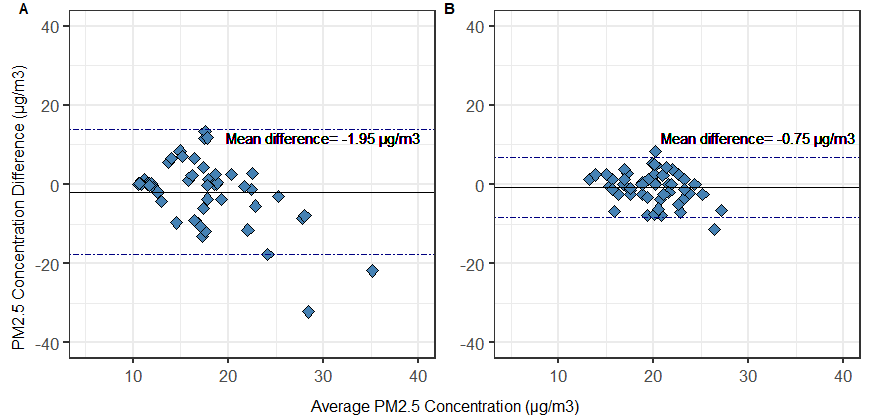


Figure S1: Bland-Altman agreement plots for the ECM (A) gravimetric and (B) nephelometric comparisons at concentrations below the 25^th^ percentile.


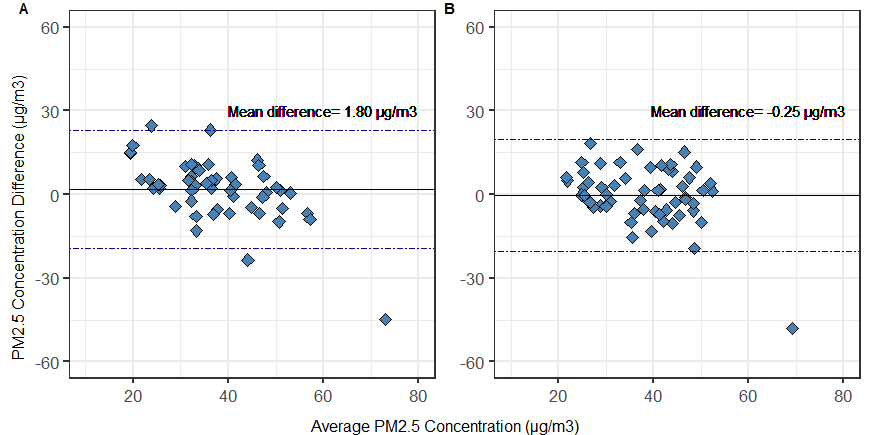


Figure S2: Bland-Altman agreement plots for the ECM (A) gravimetric and (B) nephelometric comparisons at concentrations between the 25^th^ and 50^th^ percentile.


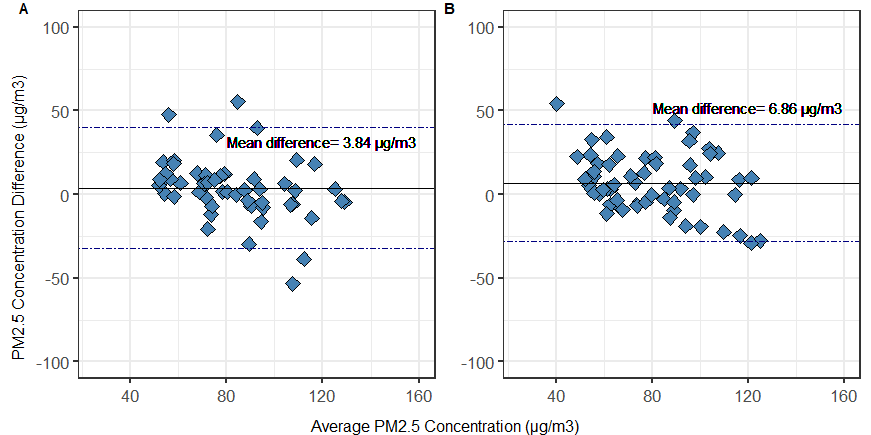


Figure S3: Bland-Altman agreement plots for the ECM (A) gravimetric and (B) nephelometric comparisons at concentrations between the 50^th^ and 75^th^ percentile.


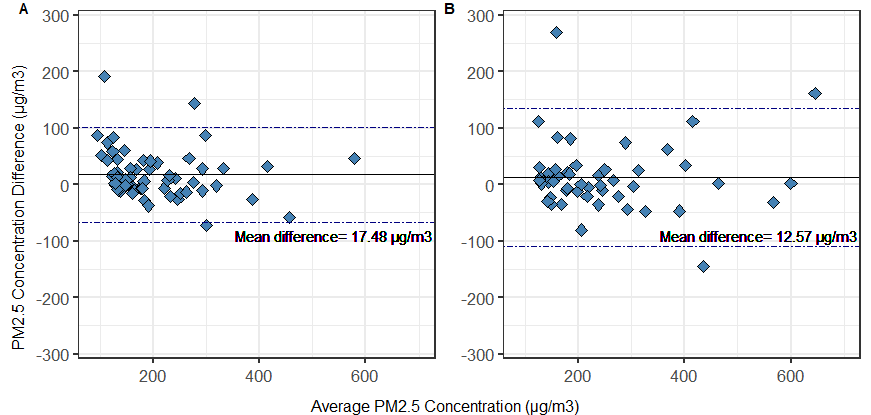


Figure S4: Bland-Altman agreement plots for the ECM (A) gravimetric and (B) nephelometric comparisons at concentrations above the 75^th^ percentile.


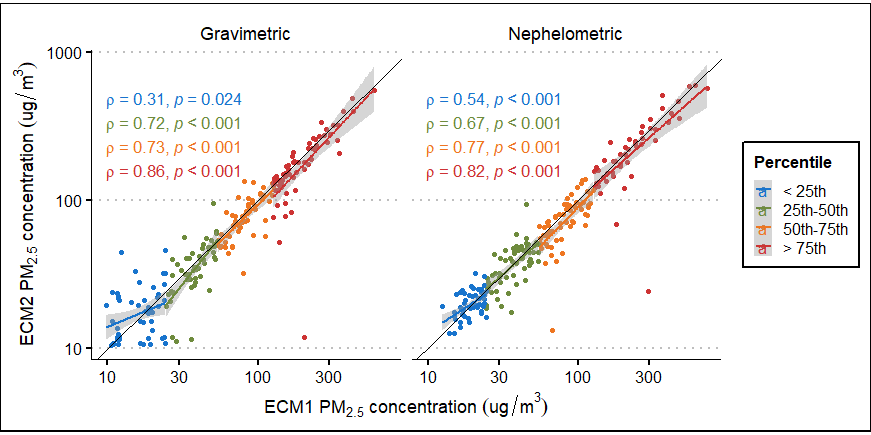


Figure S5: Correlation plots for the ECM gravimetric and nephelometric comparisons by data percentiles
